# Health outcomes in offspring of mother with breast implants: A protocol of systematic review and meta-analysis

**DOI:** 10.1097/MD.0000000000014689

**Published:** 2019-03-08

**Authors:** Ailin Song, Jie Dang, Zhiyun He, Youcheng Zhang, Xiaokang Liu, Lei Zhao, Xi Lv, Yumin Li

**Affiliations:** a VIP Surgical Department of Lanzhou University Second Hospital, Lanzhou; b Center of Children's Medical Examination, Lanzhou University Second Hospital; c Beijing Friendship Hospital of Capital Medical University, Beijing; d General Surgery Department of Lanzhou University Second Hospital, Lanzhou, China.

**Keywords:** breast augmentation, breast implant, health outcomes, meta-analysis, morbidity, offspring, systematic review

## Abstract

**Background:**

An increasing number of women undergo breast augmentation at their reproductive age. The most existing evidence focuses on the impact of breast implant on the index women's health and breastfeeding after they give birth to a child. No previous systematic review has investigated the association between breast implant in mother and health outcomes in offspring. In this study, we aimed to conduct a systematic review and meta-analysis to evaluate the influence of breast implant on offspring's health outcomes.

**Methods:**

A comprehensive search strategy will be conducted including the following databases: MEDLINE (via PubMed), Embase, Cochrane Central Register of Controlled Trials, Chinese Biomedical Literature Database, China National Knowledge Infrastructure, Wan Fang Data. The World Health Organization (WHO) International Clinical Trials Registry Platform (ICTRP) will be searched as well for retrieving the ongoing studies. The cohort study and case–control study will be considered as eligible study if investigating the impact of breast implant in mother on health outcomes in offspring. The risk of bias of included studies will be appraised by the Newcastle–Ottawa scale.

**Results:**

The results of this study will be presented in the full-text of the systematic review.

**Conclusion:**

This systematic review and meta-analysis will infer a conclusion on the association between breast implant in mother and health outcomes in offspring, and the quality of existing evidence.

**PROSPERO registration number:** CRD42019121221

## 1 Introduction

Nowadays, there are increasing number of hospitalized records on breast implant at women's reproductive age. This medical skill was only introduced in 1962, but there were more than two million women underwent breast implant in the world by 1992.^[1]^ After that the number still keeps arising. For example, the number of women having cosmetic breast augmentation increased by 1.5-folds within one decade from 2001 to 2011 in Australia, by 45% during the same period in United States, and by twofolds from 2005 to 2013 in United Kingdom.^[2]^ Although the US Food and Drug Administration asked to stop this surgery temporarily in 1992 because of limited evidence supporting no harm of silicone gel breast implant.^[3]^ Many studies about the impact of breast implant on health outcomes in women have been conducted since the requirement. However, a recent well-conducted systematic review still indicated that better evidence on this field should be produced continuously for that the current evidence cannot infer a conclusion about the effect of breast implant on subsequent adverse outcomes.^[4]^ At the meantime, the association between breast implant in mother and outcomes related to offspring has also been investigated. But most studies focused on the impact of breast implant on breastfeeding.^[2,5–7]^ The association between breast implant in mother and adverse outcomes in offspring was rarely studied at early stage until some hospitalized cases of children probably induced by breastfeeding by mother with breast implant were reported.^[8–11]^ Fortunately, several studies of which the data were from the Nordic European countries present the impact of breast implant on the rate of morbidity in offspring.^[12–14]^ Also a review including these studies was also published previously.^[1]^ Although the evidence on this association remained inconclusive due to small number of studies, limited statistical power and without quantitative meta-analysis, systematic review and meta-analyses have suggested that the women with breast implant are less likely to have exclusive breastfeeding.^[6,7]^ It is plausible that breast implant may influence offspring's health by less breastfeeding in long term, because breastfeeding has been suggested as an independent protective factor on development and health of babies and psychological health of mothers.^[15–18]^ Thus, it is necessary to conduct a comprehensive systematic review and meta-analysis to confirm whether breast implant in mother can increase the risk of long-term adverse health outcomes in offspring. In this study, we aimed to systematically evaluate the impact of breast implant on health outcomes in offspring.

## 2 Methods

The protocol of this systematic review and meta-analysis had been registered in the PROSPERO international prospective register of systematic reviews (Register number: CRD42019121221. https://www.crd.york.ac.uk/PROSPERO/display_record.php?RecordID=121221). This protocol was reported in accordant with the preferred reporting items for systematic review and meta-analysis protocol (PRISMA-P).^[19,20]^

### 2.1 Eligibility criteria

We will include the studies, if the following criteria are met: offspring born to mothers with breast implant; focusing on the investigation of the effect of breast implant on health outcome in offspring; the outcomes of interest should be rate of morbidity (including cancer, congenital malformations, esophageal disorders, rheumatic diseases, pyloric stenosis, low birth weight, neonatal intensive care, perinatal death, and preterm delivery) or breastfeeding; cohort study or case–control study. In order to minimize the missing of relevant studies, we plan to track the references of the existing related reviews, systematic reviews and meta-analyses.

### 2.2 Information source

A systematic search will be conducted, which includes the following databases: MEDLINE (via PubMed), Cochrane Central Register of Controlled Trials, Embase, Chinese Biomedical Literature Database, China National Knowledge Infrastructure and Wan Fang Data. The ongoing or unpublished studies will be retrieved by an extra search in the World Health Organization (WHO) International Clinical Trials Registry Platform (ICTRP) (http://apps.who.int/trialsearch/Default.aspx). We will check the references of included articles to retrieve further relevant studies.

### 2.3 Search strategy

We plan to perform the electronic search from the inception of databases to December 31, 2018 without any language limitation, and update the search before submitting the manuscript of full-text to peer-review journal. The search terms include all the academic terminology related to breast implant and offspring. The example of search strategy in PubMed can be seen below:

#1 “breast implants”[Mech] OR “breast implantation”[Mesh] OR “breast implant”[Title/Abstract] OR “breast implants”[Title/Abstract] OR “breast implantation”[Title/Abstract] OR “breast augmentation”[Title/Abstract] OR mammaplasty[Title/Abstract] OR mammoplasty[Title/Abstract]
#2 offspring[Title/Abstract] OR adolescent[Title/Abstract] OR child∗[Title/Abstract] OR offspring[Title/Abstract] OR “Adolescent”[Mesh] OR “Child”[Mesh] OR “Infant”[Mesh]
#3 #1 AND #2

The details of search strategy can be found in the supplement file.

### 2.4 Study selection and data extraction

After the electronic search, the records will be managed in the EndNote X7 literature management software. A pairs of researchers will independently select the potential eligible study by reviewing the title and abstract. And then the full-texts of the potential studies will be retrieved and reviewed by the reviewers separately.

A data extraction form will be developed by the research group, and includes the following items: title, first author, publication type, publication year, country, journal, the sponsor, inclusion and exclusion criteria, study design, length of follow-up, sample size, race, age, type of breast implant, lost/withdrawal, outcomes. In order to assure the high inter-rater reliability, we plan to do a pilot test of data extraction. And then a pair of researchers will independently extract the data of included studies.

When there is disagreement, it will be solved by discussion between the pair of reviewers or by a third researcher.

### 2.5 Risk of bias assessment

In this systematic review, we plan to include both cohort study and case–control study. For keeping comparability of evaluation of risk of bias between the 2 types of study, the risk of bias of included studies should be assessed by the same tool or scale. The Newcastle–Ottawa scale (NOS) for observational studies well designed and effective instrument for assessing the risk of bias of both cohort study and case–control study.^[21]^ The NOS for cohort study intend to evaluate the potential risk of bias of cohort study by the following aspects: representativeness of the exposed cohort, selection of unexposed cohort, ascertainment of exposure, demonstration on that outcomes of interest was not present at the start of study, comparability of cohorts on the basis of the design or analysis controlled for confounders, assessment of outcome, long enough of the length of follow up to observe the outcomes, and adequacy of follow up of cohorts). The NOS for case–control study has similar elements including: is the case definition adequate? representativeness of the cases, selection of controls, definition of controls, comparability of cases and controls on the basis of the design or analysis, ascertainment of exposure, same methods of ascertainment for cases and controls, nonresponse rate. Eventually, the quality of included studies will be rated as good, fair, and poor quality.

A pair of reviewers will evaluate the risk of bias of included studies independently. When meeting disagreement, solution will be figured out by discussion or consulting a third researcher.

### 2.6 Data synthesis

The meta-analysis will be performed in STATA 12.0. We will evaluate the effect estimate by pooled risk ratio (RR) and 95% confidence intervals (CI). Before synthesizing the results from different included studies, the heterogeneity between studies will be tested by *I*^*2*^. If *I*^*2*^ ≤ 50%, we will pool the data by Mantel–Haenszel fixed-effects model. But if *I*^*2*^ > 50%, we will continue to evaluate the sources of inconsistency by sub-group analysis and meta-regression. If the results indicate no evidence on clinical heterogeneity, we will pool the results by Mantel–Haenszel random-effects model. Otherwise, stratified analysis according to the clinical character will be performed, if power is enough. But data synthesis will not be performed and a description of the results of the included studies will be given instead for limited statistical power. With regard to the assessment of reporting bias, the Begg's and Egger's funnel plot method will be used.^[22,23]^ In addition, the contour-enhanced funnel plot will be used to help to distinguish asymmetry, if multifactors lead to publication bias.^[24]^

### 2.7 Quality of evidence

The Grading of Recommendation, Assessment, Development and Evaluation approach (GRADE) is an international accepted tool for assessing the quality of evidence.^[25]^ The process will be performed on the platform of GRADEpro—GDT (https://gradepro.org/). The possible grades of quality of evidence by GRADE include “high,” “moderate,” “low” and “very low” which indicate how strong confidence we have on the body of evidence. The quality of evidence from observational studies is rated as “low” primarily. The 5 factors (risk of bias, directness, inconsistency, imprecision of effect estimates, and publication bias) are considered to decide whether downgrading the quality of evidence. Once the quality of evidence has not been downgraded by any factors, we will continue to assess whether there is possibility to upgrade the quality of evidence by the 3 factors (large magnitude of effect, dose-response gradient, and plausible confounding).

### 2.8 Ethics and dissemination

There is no need to get an ethical approval and patient informed consent because this research is a systematic review and meta-analysis.

## 3 Discussion

To the best of our knowledge, there is no previous systematic review and meta-analysis investigating the association between breast implant in mother and adverse health outcomes in offspring. Our study will present whether there is relationship between the risk factor and outcomes of interest by comprehensively retrieving the relevant studies and strictly appraising the results and quality of included studies. If there is an association, a professional interpretation will also be presented.

## Author contributions

Conception and design of this systematic review and meta-analysis (Ailin Song, Jie Dang, Zhiyun He, Youcheng Zhang, Xiaokang Liu, Lei Zhao, Xi Lv, Yumin Li); tested the feasibility of the study (Ailin Song, Jie Dang); developed the search strategy (Ailin Song, Zhiyun He); drafted this protocol (Ailin Song, Jie Dang,), revised the protocol (Ailin Song, Jie Dang, Zhiyun He). All authors provided critical revisions of the protocol and approved the final manuscript.

**Conceptualization:** Ailin Song, Jie Dang, Zhiyun He, Youcheng Zhang, Xiaokang Liu, Lei Zhao, Xi Lv, Yumin Li.

**Methodology:** Ailin Song, Jie Dang, Zhiyun He, Youcheng Zhang, Xiaokang Liu, Lei Zhao, Xi Lv.

**Resources:** Ailin Song.

**Supervision:** Yumin Li.

**Validation:** Ailin Song, Jie Dang, Youcheng Zhang, Xiaokang Liu, Lei Zhao.

**Visualization:** Xi Lv.

**Writing – original draft:** Ailin Song.

**Writing – review & editing:** Ailin Song, Jie Dang, Zhiyun He.
